# Transcription start site signal profiling improves transposable element RNA expression analysis at locus-level

**DOI:** 10.3389/fgene.2022.1026847

**Published:** 2022-10-21

**Authors:** Natalia Savytska, Peter Heutink, Vikas Bansal

**Affiliations:** German Center for Neurodegenerative Diseases (DZNE), Tübingen, Germany

**Keywords:** transposable elements (TEs), TSS, k-means clustering, RNA-seq, simulation, transposons

## Abstract

The transcriptional activity of Transposable Elements (TEs) has been involved in numerous pathological processes, including neurodegenerative diseases such as amyotrophic lateral sclerosis and frontotemporal lobar degeneration. The TE expression analysis from short-read sequencing technologies is, however, challenging due to the multitude of similar sequences derived from singular TEs subfamilies and the exaptation of TEs within longer coding or non-coding RNAs. Specialised tools have been developed to quantify the expression of TEs that either relies on probabilistic re-distribution of multimapper count fractions or allow for discarding multimappers altogether. Until now, the benchmarking across those tools was largely limited to aggregated expression estimates over whole TEs subfamilies. Here, we compared the performance of recently published tools (SQuIRE, TElocal, SalmonTE) with simplistic quantification strategies (featureCounts in unique, fraction and random modes) at the individual loci level. Using simulated datasets, we examined the false discovery rate and the primary driver of those false positive hits in the optimal quantification strategy. Our findings suggest a high false discovery number that exceeds the total number of correctly recovered active loci for all the quantification strategies, including the best performing tool *TElocal*. As a remedy, filtering based on the minimum number of read counts or baseMean expression improves the F1 score and decreases the number of false positives. Finally, we demonstrate that additional profiling of Transcription Start Site mapping statistics (using a *k*-means clustering approach) significantly improves the performance of *TElocal* while reporting a reliable set of detected and differentially expressed TEs in human simulated RNA-seq data.

## Introduction

The dysregulation of Transposable elements (TEs) has been associated with many phenotypes and disorders such as ageing (Andrenacci, et al., 2020; Gorbunova et al., 2021), neurodegenerative diseases (Guo et al., 2018; Jacob-Hirsch et al., 2018; Savage et al., 2019) and cancers (Jansz and Faulkner, 2021; Grundy, et al., 2022). These findings fuel the interest in profiling the repeatome on a global scale in related or similar physiologies. For instance, TEs transcriptional profiling led to the formulation of the “retrotransposon storm” hypothesis of age-dependent neurodegeneration due to a global derepression of TEs (Dubnau, 2018). These discoveries also opened new therapeutic avenues targeting the activity of TEs by applying viral suppressants (Gold et al., 2019; Tam et al., 2019).

TEs are repetitive DNA segments that have the ability to move and replicate in the genome and occupy large fractions in mammalian genomes. At least 45% and 37.5% of the human and mouse genome, respectively, is composed of TE DNA sequences (Pace and Feschotte, 2007). Consequently, the computational analysis for the detection and differential expression (DE) of TEs face significant challenges due to a high false discovery rate (FDR). The repetitiveness of TEs leads to the generation of multiple identical or highly similar reads that can be attributed back to multiple genomic loci, i.e. multimappers. Moreover, many TEs are annotated in intronic regions, which makes it difficult to distinguish between the autonomous TE transcription and exaptation events like TE exonization in coding transcripts (Zemojtel et al., 2007; Lin et al., 2008; Schmitz and Brosius, 2011; Park et al., 2012; Davis et al., 2017; Deininger et al., 2017; Gonçalves et al., 2017). Both of these challenges can exacerbate one another, as an expression of the coding transcript with an exapted TE sequence might be reflected in multimappers. To resolve these challenges, the expression analysis of TEs at the subfamilies level has become a popular strategy. However, in some cases, the activity of a single locus could also be the main driver for “subfamily” level overexpression and hence, the primary pathology cause. Such singular active loci could promote tumorigenesis *via* the regulation of oncogenes (Babaian et al., 2016; Jang et al., 2019). Another evidence for this comes from discordant epigenetic profiles of various normal and tumour tissues, where only some TE loci were demethylated, and most of the loci within the same subfamilies remain repressed (Ewing et al., 2020).

Regardless of the quantification level (locus or subfamily), two major approaches are usually undertaken to deal with multimappers—1) incorporate them and distribute their total counts or fractions between the putative origin entities; 2) discard the multimappers altogether. Depending on the tool, the former strategy may overinflate the expression estimates for subfamilies; while the latter may result in an estimate of mappability rather than an expression for the subfamilies, especially for the evolutionary younger ones (Lanciano and Cristofari, 2020). One major strategy for incorporating multimappers in the TE expression analysis, irrespective of the analysis level (locus or subfamilies), is leveraging the Expectation-Maximization (EM) algorithm into the quantification step. This method relies on the iterative redistribution of the count fractions across putative read origin loci/consensus sequences, which may [SalmonTE (Jeong et al., 2018), SQuIRE (Yang et al., 2019)] or may not [TEtranscript (Jin et al., 2015), TElocal (https:/github.com/mhammell-laboratory/TElocal)] include the number of uniquely mapping reads in the estimation for each entity. Inclusion or exclusion of unique mappers depends on the basic underlying assumption about TE-derived reads—1) loci/subfamilies that already have unique mappers unambiguously derived from them are more likely to be the source for the multimappers (SQuIRE, SalmonTE); 2) younger subfamilies and their individual copies (loci) have higher similarity and hence will be primarily represented by multimappers (TEtranscript, TElocal). While benchmarking across the publicly available tools is previously reported (Teissandier et al., 2019; Schwarz et al., 2022), an effort to propose a systematic downstream strategy for improving the performance of existing methods is still lacking, particularly at the loci level.

In this short report, we evaluated 8 TE quantification pipelines (4 tools) based on simulated (synthetic) RNA-seq data from human and mouse. We tested the most recent and popular quantification strategies with a focus on locus-specific detection and differential expression in a dataset simulated for both gene and TE expression. Hence, we included featureCounts within Rsubread (Liao, et al., 2014), SalmonTE, TElocal within the TEToolkit suite and SQuIRE. We aimed to identify the best performing tool using simulated RNA-seq data, subsequently improving the FDR and F1 score by utilising the mapping statistics around the Transcription Start Site (TSS) of TEs. Among the considered tools here, TElocal produced the best results based on our simulated RNA-seq data. We demonstrated that the choice of counts and baseMean expression cutoff is critical for reducing the false positive hits. Furthermore, *k*-means clustering based on the signals around the TSS of TEs aided us in filtering out a substantial amount of false positives. In a nutshell, we propose an additional TSS profiling downstream of TElocal along with visual inspection of genomic regions to significantly improve the TE expression analysis at the loci level.

## Materials and methods

### Bulk RNA-seq data simulation

Both stranded and unstranded paired-end 100-bp long reads were simulated from the human substantia nigra (∼37 million reads) and the mouse forebrain (∼47 million reads) using R (v.4.0.3) and RStudio (v.1.3.1093). R package polyester v.1.26.0 (Frazee et al., 2015) was used for simulating RNA-seq data, derived from both TEs and genes. For additional details see Supplementary Methods.

### Tested strategies and tools

We chose the widely accepted strategies for TEs quantification that allowed for the quantification on the locus level [e.g. SQuIRE (v.0.9.9.92) and TElocal (v.0.1.0)] or allowed for building a custom library [SalmonTE (v.0.4)]. We excluded the tools that at the moment of benchmarking had limited use restricted to single class of elements and no custom database building functionality [REdiscoverTE (Kong et al., 2019)] and/or did not get developers support at the time [Telescope (Bendall et al., 2019)].

In addition to EM-mode, SQuIRE and TElocal have a unique mode as an additional quantification option that we also tested. SalmonTE relies on the quasi-mapping with Salmon for which custom databases were built using default command “index” and fasta file containing all TE instances [from mm9 (mouse) and GRCh38 (human) reference genomes]; EM step applies to the sets of reads mapping to the identical sets of target TEs. However, SalmonTE does not have a specific strategy to deal with the reads that span both gene and TE. TElocal and SQuIRE rely on the RNA-seq read alignment, e.g. produced by STAR; SQuIRE by design relies on mapping with STAR, hence we chose STAR (v.2.7.5a) (Dobin et al., 2013) and used the same alignment files for quantification. SQuIRE and TElocal prioritise read assignment to genic coding regions over the TEs to account for genic reads incorporating TEs within them.

In addition, three simplistic quantification strategies were assessed using featureCounts function of rSubread R package (v.1.34.7), which relies on the provided alignment files. We leveraged the inbuilt strategies of dealing with multimappers i.e. exclude multimappers, distribute fractions of counts evenly or assign randomly.

### Transposable element detection benchmarking

The main parameters we relied upon for comparing the performance of different strategies were FDR and F1 score. Identified TEs were considered True Positive, if they were both actually present in the simulation and they were assigned more counts than the detection thresholds tested—0, 5, 10, 20, 30, 50, 70, and 100 raw counts. For human stranded simulation, we also tested the length filters, which would exclude elements below 50, 100, 150, 200, 250 or 300 bp. Detection filtering cutoff used raw counts assigned per element after mapping the reads.

### Transposable element differential expression benchmarking

We used FDR and F1 score for assessment of differential expression of TEs (DE-TEs). Identified DE-TEs were considered True Positive, if they 1) were simulated to be DE, 2) had the correct direction of the expression change as the simulated one, and 3) had a baseMean expression above a filter threshold, 4) had padj <0.01 and |log2FC| ≥2. For human simulation, we also tested the length filters. Differential expression detection used DESeq2 normalized baseMean values for filtering after running DE analysis.

### Transcription start site profiling

We estimated the coverage for a window of 400 bp around TSS and used it to further split putatively active TEs into True or False Positives. Coverage was calculated for alignment files using deeptools (v.3.5.1) (Ramírez et al., 2016) bamCoverage (“--binSize 1 --normalizeUsing RPKM”). We used all putatively active loci passing the threshold of five counts irrespective of their status (True or False) and further aggregated coverage statistics with deeptools computeMatrix (“--beforeRegionStartLength 200 --referencePoint TSS”). *K*-means clustering (“--silhouette”) was further applied to the coverage statistics matrix using deeptools plotHeatmap. Silhouette score is a metric to study the goodness of a clustering technique. The silhouette ranges from −1 to +1, where a high value indicates that a region is well matched to its own cluster. *K* between 3 and 8 were tested for optimising the number of filtered FP hits using average silhouette scores, F1 score, and percentages for FP and TP. Visual inspection of cluster’s TSS profiles was used to assign the clusters between True and False categories. If a reliable peak of averaged coverage coincided within the window downstream of TSS, the cluster was assigned as True; if coverage was even or peaked upstream to TSS the cluster was assigned as False. *K* = 2 was excluded both due to its insufficiency to differentiate between our minimal expectation of three different TSS profiles shapes described above, as well as its confirmed poor performance for the trial sample. FN was assessed as a number of actual TP in False clusters. FDR and F1 scores were calculated and compared to the performance statistics of the expression-based filtering. Only the TEs in the identified True clusters were further retained for the DE analysis for the calculation of the FDR. This was achieved by assigning 0 counts per sample to those elements, which were outside the True clusters. The further quantitative and qualitative estimates were obtained as described in the previous method section.

## Results

**FIGURE 1:**
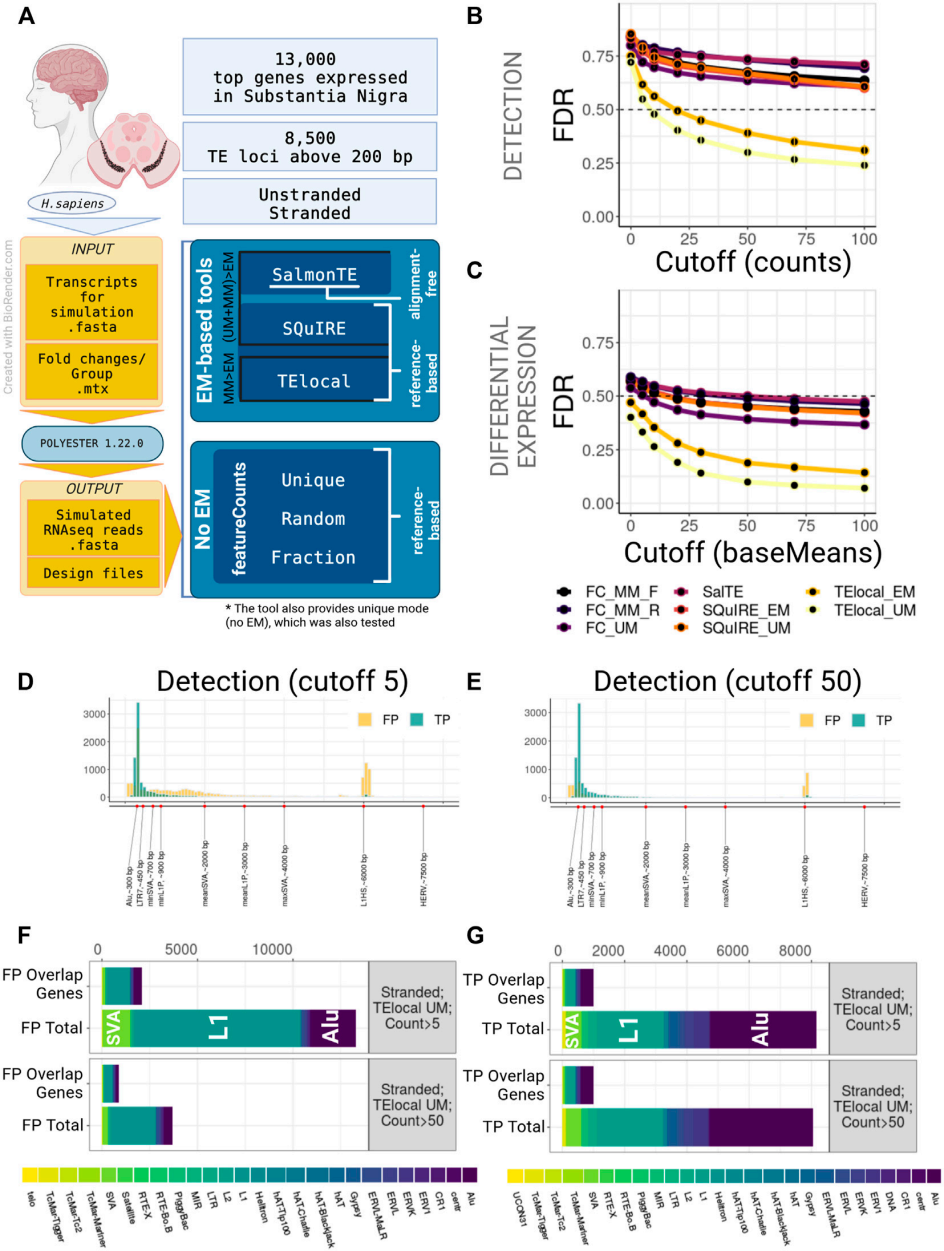
Benchmarking of TE quantification tools on human model, stranded experiment. **(A)** A general overview of the simulation setup and the strategies used in benchmarking. For human simulation 2,000 TEs over 200 bp long and top 13,000 genes expressed in substantia nigra were simulated in stranded and unstranded experiments using Polyester 1.22.0. The resulting simulated sequencing data was processed using 3 EM-based tools (both in EM and no EM modes, where permissible) and 3 modes of featureCounts. **(B)** TE Detection FDR for different detection cutoffs using the tested tools. TElocal in unique mode (TElocal_UM) outperformed other strategies closely followed by TElocal in EM mode (TElocal_MM), however even with higher cutoffs FDR reached 26%. **(C)** TE Differential Expression detection FDR for different expression cutoffs. **(D,E)** Length distribution of True Positive (TP) and False Positive (FP) hits for TElocal in UM mode at detection cutoff 5 **(D)** and 50 **(E)**. **(F)** Family Composition of total FP hits (Total) and FP hits overlapping the simulated expressed genes (Overlap Genes) for TElocal_UM at detection cutoffs 5 and 50. Only a minority of the FPs at both cutoffs can be explained by misattribution of the genic reads (2091/13301 for cutoff = 5 and 887/3705 for cutoff = 50). **(G)** Family Composition of TP hits categorized by total and overlap genes. FC_MM_F, featureCounts using multimappers in “fraction” mode; FC_MM_R, featureCounts using multimappers in “random” mode; FC_UM, featureCounts using unique mappers only; SalTE, SalmonTE; SQuIRE_EM, SQuIRE in EM mode; SQuIRE_UM, SQuIRE in unique mode; TElocl_EM, TElocal in EM mode; TElocal_UM, TElocal in unique mode.

To determine the performance of Transposable Elements (TEs) quantification pipelines, we commenced our study by simulating both stranded and unstranded paired-end sequencing reads for the human substantia nigra and the mouse forebrain (see Methods, Figure 1A; Supplementary Figure S1A; Supplementary Table S1). We assessed the detection and differential expression of TEs (DE-TEs) performance of all the pipelines using false discovery rate (FDR) and F1 score.

### Improvement of transposable elements characterization using expression thresholds

We observed that all the pipelines employed in this study, when using default parameters, performed poorly due to the high number of false positives (FPs) for both stranded and unstranded human RNA-seq data (Figures 1B,C; Supplementary Figure S2B). For instance, using human stranded simulated data, without applying any filtering to the putatively active TEs, we found a FDR range of 71%–86% and 40%–59% for detection and DE-TEs, respectively (Figures 1B,C; Supplementary Table S1A). When we applied a detection cutoff based on the counts (for detection) or baseMean (for DE-TEs), FDR decreased for all the pipelines. While *TElocal* surpassed the other methods, *TElocal* in unique mode (*TElocal_UM*) exhibited the lowest FDR value followed by *TElocal* in multimapping mode (*TElocal_MM*).

Next, we focused on the characterization of the FP hits using several strategies to mitigate their numbers in the best performing pipeline (*TElocal_UM*). We hypothesised that the two major sources of the FPs can be related to either mapping errors or annotation errors. To test their relevance, we focused on characterising FP content, length distribution and overlap with the simulated genes. For human simulation and detection with *TElocal_UM*, we observed a presence of shorter elements in FPs with the length below the minimal cutoff for the simulated elements (TPs) length (Figures 1D,E; Supplementary Figure S2C). Additionally, we observed much higher enrichment for longer elements in FPs compared to TPs—a secondary peak at 6 kbp that corresponds to the average length of L1HS elements. We then tested TE length as a filter in order to reduce the FPs that possibly arise from the misattribution of reads to the shorter elements (Supplementary Figures S2A,E), nevertheless, FDR did not significantly improve for either of the detection cutoffs (5 and 50 counts or baseMean). When the composition of FP hits for both cutoffs (5 and 50 counts, 200 bp length) was examined, L1 elements were revealed to be the dominating FPs, followed by Alu and SVA elements (Figure 1F; Supplementary Figure S2D). The general composition of the FP hits at the detection cutoff five was strongly enriched in L1P and L1HS elements, as well as SVAs (Supplementary Tables S2A,B). While expression was simulated for only 491 SVA loci for stranded data, *TElocal_UM* detected 1455 SVA loci as putatively active, including 67 loci for SVA_B that had no simulated loci and 911 loci for SVA_D that had only 4 loci simulated. Similarly, far fewer loci for L1 elements were simulated as compared to the number of loci detected as putatively active. Only a minority of the total FPs (16%–24% and 18%–29% for stranded and unstranded simulations, respectively) could be explained by the reads misattribution derived from the overlapping simulated genes (Figure 1F; Supplementary Figure S2D). Similar distribution was observed for the TPs (Figure 1G).

We obtained similar results for the mouse simulation RNA-seq dataset (Supplementary Figure S2A; Supplementary Table S1B). Poor FDR performance for all the pipelines was improved upon the increase of the detection threshold, with the *TElocal_UM* outperforming other methods (Supplementary Figures S1B,E; Supplementary Tables S3E,F). Lower cutoffs were required to achieve major FP reduction, as compared to the detection of the human simulation. Majority of the mouse FP hits at the detection level were driven rather by long TEs of L1 and ERV classes with very few short elements (Supplementary Figures S1C,D,F,G). Detection of the short SINE elements such as B2 and B4 was impaired with the best performing pipeline, suggesting a possible bias in detecting these elements (Supplementary Figures S1D,G). Consistent with the human simulation, only a minor fraction of FP hits could be explained by the misattribution of reads derived from the simulated genes (∼9% of FP hits at cutoff 5).

### Improvement of transposable elements characterization using transcription start site profiling

**FIGURE 2:**
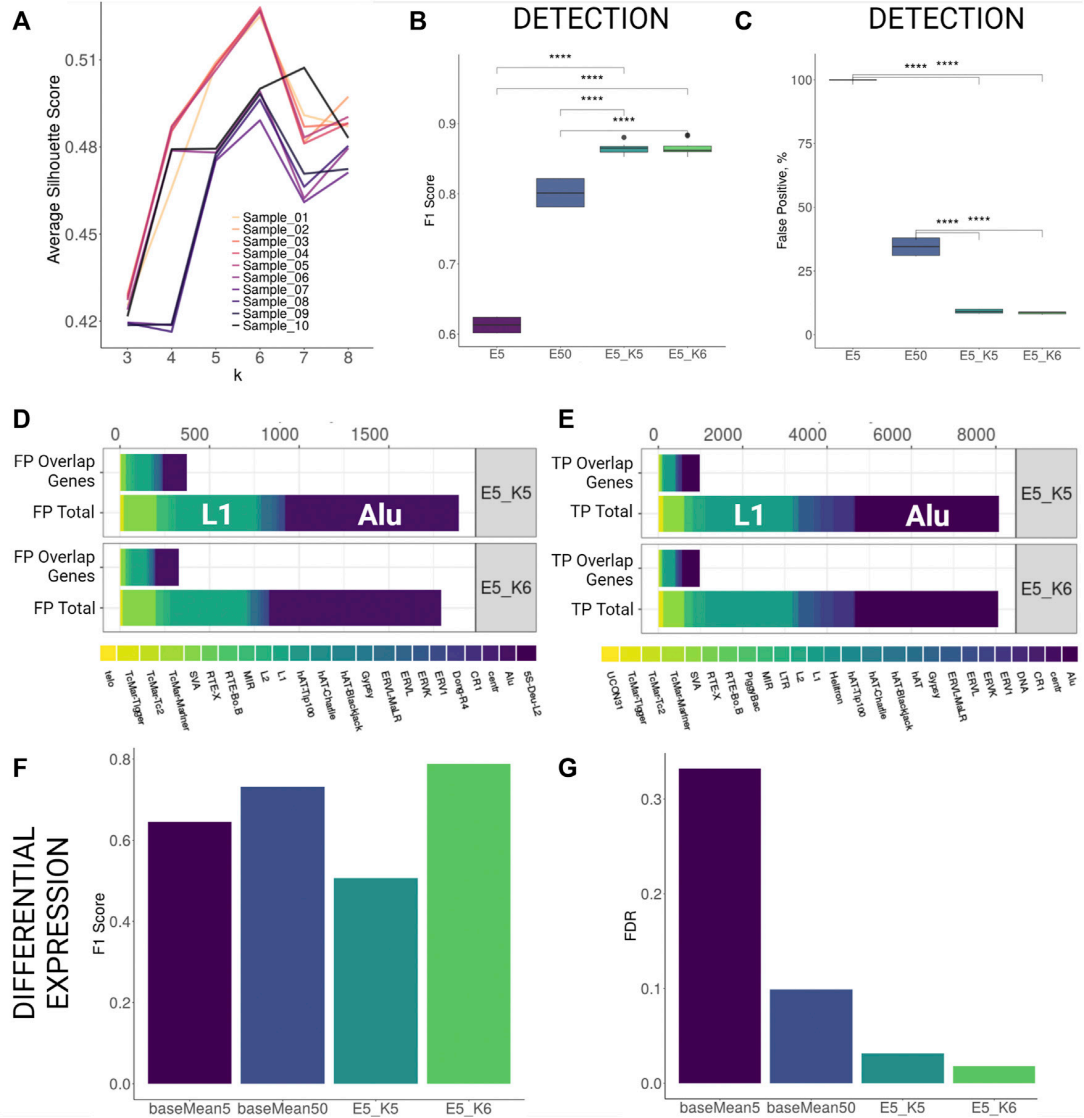
TSS profiling improves detection and differential expression detection for human stranded simulation. **(A)** Average silhouette scores for different *k* values per each sample. Best silhouette scores for most samples are reached with *k* = 5 and *k* = 6. **(B,C)** F1 Score and False Positive proportions improvement with TSS profiling at the detection level. Both parameters improve significantly (Wilcoxon signed-rank test, *p* < 0.001) when TSS profiling is applied to elements retained with detection cutoff 5 (E5_K5 and E5_K6) as compared to only filtering by low detection cutoff (5 counts, E5) or increasing detection cutoff (50 counts, E50). **(D,E)** Family Composition of FP and TP hits categorized by total and overlap genes after TSS profiling **(F,G)** F1 Score and FDR improvement for differential expression detection with TSS profiling.

As the majority of FP hits were potentially derived from mapping errors, we aimed to profile mapping statistics over putatively active elements as a means to filter out false hits. TEs vary greatly in size, therefore, we focused on the profiling of the window of 200 bp up- and downstream of TSS (see Methods). This strategy would also potentially allow to account for the FPs derived from reads misattribution from the longer transcripts to the exapted TEs within them. The theoretical profile of the autonomously expressed (defined here as independently expressed as opposed to exapted TEs) TE elements would be reflected in a mapping peak downstream of their annotated TSS. Exapted TEs within a longer transcript element would have an evenly distributed coverage both upstream and downstream of its annotated TSS or a minor drop off in coverage down TSS at the highly repetitive regions. In the case of the erroneous mapping of reads derived from the related element, we expect coverage to be shallow and scarce across the examined locus. Hence, it is theoretically possible to separate false hits from the truly independently expressed elements based on their mapping statistics from RNA-seq data. To this end, we used *k*-means clustering to keep the clusters of TEs that showed an enrichment downstream of the annotated TSS. For instance, an enrichment profile using all the detected TEs from the human stranded simulated RNA-seq data showed a mapping peak at downstream of the annotated TSS (Supplementary Figure S3). To separate the background clusters (potentially FPs), we first assessed silhouette scores for *k* = [3:8] to find the optimal value for *k*-means clustering applied to the TSS profiles (Figure 2A; Supplementary Figure S4A; Supplementary Figures S5A,F). Based on this, we chose two best k values for downstream filtering, five to six and seven to eight for human and mouse simulation, respectively.

Using *k*-means profiling on human stranded simulation data, we observed that the detection performance improved greatly. The FDR values dropped from 54.9% (counts cutoff 5) to 11.2% (counts cutoff 5, *k* = 5) and 10.6% (counts cutoff 5, *k* = 6). This improvement was achieved by a reduction of the number of FPs by over 10 times without losing much of TP hits as the F1 score increased significantly from 61% (counts cutoff 5) to 86% (counts cutoff 5, *k* = 5) and 87% (counts cutoff 5, *k* = 6) (Supplementary Table S3C; Figures 2B,C). Importantly, the reduction in FDR using the *k*-means approach at a cutoff of 5 was ∼3-fold less than FDR at a cutoff of 50 when the *k*-means approach was not utilised (29.9). Comparing family composition of FPs and TPs before TSS profiling (Figures 1F–G) and after TSS profiling (Figures 2D,E) revealed that the TSS profiling reduces false positive TE hits irrespective of gene overlap and without losing TPs. We further tested the outcomes of the DE analysis performed on the putatively active TEs present within filtered clusters only. We found that the TSS profiling significantly reduced the FDR (*k* = 6, FDR = 1.7%) and increased the F1 score (*k* = 6, F1 = 0.79) as compared to using only a baseMean expression filter of 5 (FDR = 33%, F1 = 0.61) and 50 (FDR = 10%, F1 = 0.73) (Figure 2F,G; Supplementary Table S3I). We then compared the expression levels of the differentially expressed elements detected with only high expression cutoff against TSS profiling approach. The application of TSS profiling allowed for detecting some lowly expressed elements, which would be otherwise filtered out with a higher expression cutoff. However, overall mean expression of the detectable elements was higher after TSS profiling (Supplementary Figure S3D). Similarly, detection was improved for human unstranded simulation i.e. FDR dropped from 58.7% (counts cutoff 5) and 37.5% (counts cutoff 50) to 11.2% (counts cutoff 5, *k* = 5) and 10.5% (counts cutoff 5, *k* = 6) (Supplementary Figures S4B,C; Supplementary Table S3D). TSS profiling also improved DE detection FDR (*k* = 6, FDR = 29%) as compared to applying a low expression cutoff (baseMean = 5, FDR = 35%), however, the profiling did not outperform the performance when a higher expression cutoff (baseMean = 50, FDR = 14%) was applied (Supplementary Figures S4D,E; Supplementary Table S3J).

Application of the TSS profiling and clustering as a filtering measure improved the detection outcome for the simulated stranded RNA-seq data from mice (FDR = 18.1% at *k* = 7 as well as *k* = 8), when compared to the basic detection cutoff of 5 (FDR = 60%). However, the results were comparable to the higher expression cutoff filter (counts cutoff 50, FDR = 18.4%) (Supplementary Figure S5C; Supplementary Table S3G). Additionally, the F1 score also improved with TSS profiling (F1 = 0.88 at counts cutoff five and *k* = 7 or 8) or high detection cutoff (F1 = 0.88 at counts cutoff 50) as compared to the basic detection cutoff (counts cutoff 5, F1 = 0.55 Supplementary Figure S5B; Supplementary Table S3). At the level of detecting DE-TEs, TSS profiling was outperformed by filtering with the high expression cutoff (baseMean = 50, FDR = 5.4%, F1 = 0.85) (Supplementary Figure S5D; Supplementary Table S3K). Similar to what we observed for the human stranded simulation, TSS profiling allowed us to detect some lowly expressed TEs, and the overall mean expression of these elements was higher than the elements retained with the high expression cutoff filtering (Supplementary Figure S5E). Finally, we obtained comparable results for the simulated unstranded RNA-seq data from mice (Supplementary Figure S5F–I; Supplementary Table S3H,L).

## Discussion

Detecting the expression of TEs at the individual loci level remains a challenging task that includes the choice of methods, parameters and downstream filtering criteria. To resolve this, we first performed benchmarking of various quantification strategies using simulated short-read RNA-seq data on humans and mice. In general, all the pipelines used in this study performed poorly if no filtering was applied. TElocal is compared favourably to the other methods and worked slightly better for stranded paired-end reads than unstranded paired-end reads. Filtering on the minimum number of read counts is an important parameter to consider as we see a significant decrease in the number of false positives by increasing the mapped reads cutoff. While exploring the source of false positives, we found that only a small fraction of the TE false positives overlapped with the simulated gene coordinates. Low rates of false positives that could be derived from genic reads and high rates for unsimulated loci presence in the putatively active dataset suggest that most false positive hits might be derived from erroneous mapping. Following these leads, we observed that such errors directly lead to false identification of whole subfamilies of elements as active, affecting the analysis quality on the whole subfamily level. We did not find any significant relation between the false positive hits and the length of TEs. However, there was an overrepresentation of L1HS loci identified as false positives. It has been previously proposed that younger active mobile elements have relatively fewer variants, which makes them challenging to characterise with current technology on the individual loci level (Criscione et al., 2014; Jin et al., 2015). As shown before (Teissandier et al., 2019; O’Neill et al., 2020), specifically younger L1 elements in the human genome have one of the worst mapping rates of all TEs examined in human repetitome when short-read paired-end sequencing is applied. To further reduce the false positive hits, we profiled TSS mapping signals using the *k*-means clustering approach. In the past, *k*-means clustering has been used on TSS mapping chromatin or expression data to identify ubiquitous and/or tissue-specific patterns (Shimokawa et al., 2007; Cui et al., 2017). We hypothesised that the false positive and true positive TEs would have different patterns of mapping signal distribution around the TSS; therefore, we aimed to separate them using a *k*-means clustering approach. Indeed, for the human genome assembly, our results showed a significant decrease in false positive hits and an increase in overall F1 score, especially using stranded human simulated RNA-seq data (F1 > 85%). However, the *k*-means clustering approach failed to significantly improve the results in the mouse assembly as we obtained a F1 score similar to simply increasing the count or baseMean expression cutoff. This result was not surprising as the mouse genome is known to be more permissive for retrotransposition in comparison to humans; therefore, it has a relatively complex TE landscape and annotations than the human genome (Bourque et al., 2018). This finding, however, suggests one needs to adopt the stringency of filtering parameters depending on the repetitive complexity of the organism.

Our study has some limitations due to the use of simulated RNA-seq data and the availability of limited resources. First, we relied only on one tool for simulation. We cannot exclude the possibility that multiple simulations using different tools could lead to inconsistent results. For example, a recent study used the same R package *polyester* (although a different version) for simulating most of their RNA-seq reads (Schwarz et al., 2022). The authors simulated only the TE loci but in high numbers, whereas we simulated the reads for both genes and TEs with the majority of reads derived from the genes rather than TEs to incorporate more biological information. In contrast to our results, they found SalmonTE as the best performing tool for the detection, quantification and DE of TEs. Notably, the authors relied on modifying TEtranscript to quantify TEs at the loci level instead of using TElocal explicitly, which in principle should produce the same results. Another possible limitation of our study might be that we employed only one clustering method. In the future, it would be essential to compare the performance of other clustering algorithms (e.g. Hererichal and Fuzzy C-means clustering) around the different window sizes of TSS in numerous species. Therefore, we recommend adapting the specific parameters according to the organism that is being studied and their respective repetitome qualities, such as transpositional activity, TE age and dominating TE species, as well as the expected proportion of TE transcripts in the whole transcriptome. Nevertheless, in agreement with the previous study (Schwarz et al., 2022), we observed that the slight modifications like stringent filtering cutoff of counts or baseMean expression could improve the outcome of existing methods, especially using human stranded paired-end RNA-seq data. Additionally, we showed that the TSS profiling of TEs significantly reduced the number of false positives. While further work is required to automate a robust pipeline, we envision that our study will serve as a reference guide to improve the TE expression analysis at the loci level Liao et al., 2019.

## Data Availability

The datasets presented in this study can be found in online repositories. The names of the repository/repositories and accession number(s) can be found in the article/Supplementary Material.”
