# Supplementary material for: Transcription start site signal profiling improves transposable element RNA expression analysis at locus-level
Source: Front Genet. 2022 Oct 21;13:1026847. doi: 10.3389/fgene.2022.1026847 (PMC9633680; doi:10.3389/fgene.2022.1026847)
Supplement: Supplementary file 3 [file DataSheet1.PDF]

Supplementary Methods

Simulations

The process for the mouse and human was slightly different. For both mouse and human designs, expression values were modelled as a function of length of the gene or TE as suggested by the authors of Polyester. For mouse simulation expression value for both genes and TEs was set as round(Length/2). For human simulation, expression value was defined as round(random(5 to 20)*Length/100)), hence it also relied on a random value between 5 and 20 to model differences in the intrinsic expression of the genes and elements, rather than rely on their length solely.
Fold changes for mouse simulation were also modeled to be more pronounced (so that theoretically they would be an easier case to detect) ranging between 5 and 16 and picked for each differentially expressed locus randomly. For human simulation, fold changes ranged between 2 and 8 and were also picked randomly. While our simulation aimed to model biological reality as closely as possible (e.g. inclusion of lncRNA and other genes), it is still a somewhat simplified model to assess base performance of the analyzed tools. Hence, we did not simulate and did not aim to test detection of subtle differential expression (fold changes below 2) by the tested tools.
For gene simulation in human, we downloaded GTEx (v.8) (GTEx Consortium 2013) mean TPM count table; and used the gene list for subsetting the gencode v.38 (Frankish et al. 2019) coding and lncRNA transcriptomes. This was done to ensure our dataset for simulation would also contain lncRNAs, which are known to often contain embedded TE sequences (Fort, Khelifi, and Hussein 2021) and thus can be potential drivers of the false TE expression signal. Of the simulated genes 10500 had no expression changes and 2500 genes were set to be differentially expressed. Independently of this, we chose 8500 TE loci to simulate; for 3514 loci, we simulated no changes in the expression between the two groups (TableS1 A). Active loci in subfamilies L1PA2, HERVK, SVA_F, AluYa, AluSg4, AluSx3, AluJr and AluSc8 were simulated to be overexpressed (up-regulated) in the Group B, while active loci in the subfamilies L1HS, L1PA6, L1PA8, HERVH, SVA_E, SVA_A, AluYb8, AluYb9, AluSx1 were simulated to be overexpressed in the Group A. We also simulated differentially expressed TE locus or multiple loci with opposite directions of expression changes within the same subfamily to simulate context-specific events (e.g. expression depending on flanking sequences rather than global subfamily dysregulation).
For mouse simulation, we used the top 11446 genes expressed in mouse forebrain from the Encode phase 3 (Luo et al. 2020) (https://www.encodeproject.org/), experiment ID ENCSR080EVZ. 2000 genes were set to be differentially expressed. A number of TE subfamilies previously reported as active in mouse (Molaro et al. 2014a) were chosen for the simulation; 6000 loci were simulated to be expressed, of which 2729 loci had no changes in expression and 3271 were set to be differentially expressed between the two groups (TableS1 B).
If the gene had multiple transcripts annotated, we used the longest transcript for the simulation. For TE simulation we chose from subfamilies previously described as active (Molaro et al. 2014b; Hancks and Kazazian 2012; Huang, Burns, and Boeke 2012) or relatively recent in evolutionary history of the model species (as more probable to retain transcriptional activity even if transposition is impaired) (Roy-Engel et al. 2002; Grow et al. 2015; Göke et al. 2015; Wang et al. 2014; Santoni, Guerra, and Luban 2012; Guo et

al. 2014). To further approximate the simulation to the biological reality, we chose to simulate expression only for the TE loci exceeding 200 bp length as they were more likely to still contain active promoters.
To simplify simulation and analysis process, elements present in the unplaced scaffolds and chrY were not considered for the simulation.
We simulated two condition groups for each simulation (referred to as CTRL and DIS for mouse, or A and B for human; TableS1) with 5 replicates in each, which we referred to “sample01”,”sample02” etc.
Both unstranded and stranded modes of RNA-seq were simulated to assess performance differences for the tools between the two modes. While stranded simulation is expected to yield more reliable and unambiguous mapping of the short reads, unstranded sequencing experiments are still performed due to economical reasons, as well as there exist multiple publicly available RNA-seq unstranded datasets for different model species and conditions, which may potentially be of interest to investigators of TE transcriptional activity. Results of our assessment could also be used as a guideline for future experimental design.
We used the same fold change parameters and expression parameters for respective pairs of stranded and unstranded simulations to assess how strandedness specifically impacts the analysis. As expected, we see a decrease in performance of all tools for unstranded human simulation compared to stranded human simulation. Strandedness of the experiment also affected efficiency of the proposed TSS profiling method - while simple detection could still be improved for the human simulation, differential expression detection improvement was impaired. We suggest this being due to the higher number of erroneously mapping reads and multimappers. Such mapping issues can potentially impact the relative expression estimates for specific loci, resulting in the loss of the identifiable DE signal.

TElocal custom index construction

As we excluded unplaced scaffolds and chrY from the simulation and analysis, we generated a custom index for TElocal (v.0.1.0). This version of TElocal allowed for supplying TE .gtf file for the analysis, rather than relying on the prebuilt distributed indices. We edited the main script to save the temporary index file built from .gtf. Edited script and distribution of TElocal v.0.1.0 can be found in the following GitHub repository: https://github.com/savytskanatalia/Simulation_Manuscript .

Statistical Calculation

All statistical calculations were performed using R and RStudio except for the silhouette score calculations (see below). F1 Score was calculated as a function of precision and recall with the formula 2*Precision*Recall/(Precision+Recall). We provide TE detection metrics in a sample-wise fashion (TableS3); for Differential Expression Detection single value is provided per cutoff as the single group-wise comparison is performed. Silhouette scores were calculated within deeptools run and equals averaged silhouette scores for all clusters per specific k. This average score per k was used for plotting and comparison of the scores for all k to choose the best performing k.

Fort, Victoire, Gabriel Khelifi, and Samer M. I. Hussein. 2021. “Long Non-Coding RNAs and Transposable Elements: A Functional Relationship.” *Biochimica et Biophysica Acta,*

Molecular Cell Research 1868 (1): 118837.
Göke, Jonathan, Xinyi Lu, Yun-Shen Chan, Huck-Hui Ng, Lam-Ha Ly, Friedrich Sachs, and Iwona Szczerbinska. 2015. “Dynamic Transcription of Distinct Classes of Endogenous Retroviral Elements Marks Specific Populations of Early Human Embryonic Cells.” *Cell Stem Cell* 16 (2): 135–41.
Grow, Edward J., Ryan A. Flynn, Shawn L. Chavez, Nicholas L. Bayless, Mark Wossidlo, Daniel J. Wesche, Lance Martin, et al. 2015. “Intrinsic Retroviral Reactivation in Human Preimplantation Embryos and Pluripotent Cells.” *Nature* 522 (7555): 221–25.
GTEx Consortium. 2013. “The Genotype-Tissue Expression (GTEx) Project.” *Nature Genetics* 45 (6): 580–85.
Guo, Hongshan, Ping Zhu, Liying Yan, Rong Li, Boqiang Hu, Ying Lian, Jie Yan, et al. 2014. “The DNA Methylation Landscape of Human Early Embryos.” *Nature* 511 (7511): 606–10.
Hancks, Dustin C., and Haig H. Kazazian Jr. 2012. “Active Human Retrotransposons: Variation and Disease.” *Current Opinion in Genetics & Development* 22 (3): 191–203.
Huang, Cheng Ran Lisa, Kathleen H. Burns, and Jef D. Boeke. 2012. “Active Transposition in Genomes.” *Annual Review of Genetics* 46 (1): 651–75.
Luo, Yunhai, Benjamin C. Hitz, Idan Gabdank, Jason A. Hilton, Meenakshi S. Kagda, Bonita Lam, Zachary Myers, et al. 2020. “New Developments on the Encyclopedia of DNA Elements (ENCODE) Data Portal.” *Nucleic Acids Research* 48 (D1): D882–89.
Molaro, Antoine, Ilaria Falciatori, Emily Hodges, Alexei A. Aravin, Krista Marran, Shahin Rafii, W. Richard McCombie, Andrew D. Smith, and Gregory J. Hannon. 2014a. “Two Waves of de Novo Methylation during Mouse Germ Cell Development.” *Genes & Development* 28 (14): 1544–49.
Roy-Engel, Astrid M., Abdel-Halim Salem, Oluwatosin O. Oyeniran, Lisa Deininger, Dale J. Hedges, Gail E. Kilroy, Mark A. Batzer, and Prescott L. Deininger. 2002. “Active Alu Element ‘A-Tails’: Size Does Matter.” *Genome Research* 12 (9): 1333–44.
Santoni, Federico A., Jessica Guerra, and Jeremy Luban. 2012. “HERV-H RNA Is Abundant in Human Embryonic Stem Cells and a Precise Marker for Pluripotency.” *Retrovirology* 9 (December): 111.
Wang, Jichang, Gangcai Xie, Manvendra Singh, Avazeh T. Ghanbarian, Tamás Raskó, Attila Szvetnik, Huiqiang Cai, et al. 2014. “Primate-Specific Endogenous Retrovirus-Driven Transcription Defines Naive-like Stem Cells.” *Nature* 516 (7531): 405–9.

Supplementary Figures

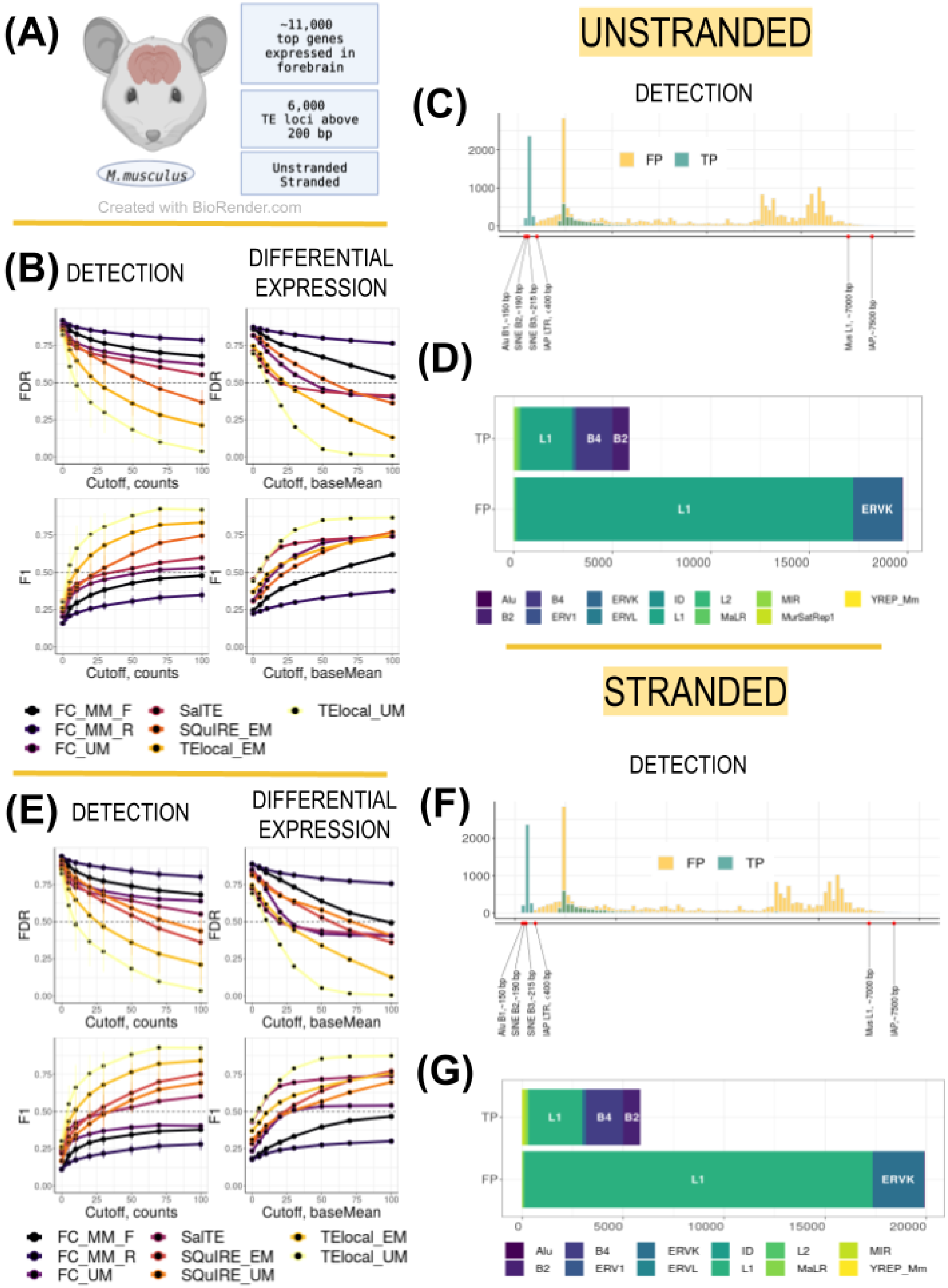

Figure S1. Benchmarking of TE quantification tools on mouse unstranded (B-D) and stranded (E-G) model. (A) - Mouse Simulation setup diagram - RNA-seq experiment was simulated using top ~11000 top genes expressed in mouse forebrain and 6000 TE loci from known active subfamilies and with length above 200 bp. (B, E) - FDR and F1 score for detection and differential expression detection by all examined tools and strategies at 8 different expression cutoffs for mouse unstranded (B) and stranded (E) simulation data. Both metrics can be improved for each tool with the increase of the detection threshold, however TElocal in unique mode (TElocal_UM) outperformed other competitors. Raw counts cutoffs were applied (counts) for detection testing and improvement; DESeq2 normalized counts (baseMean) were applied for differential expression testing and improvement. (C-D), (F-G) - Length and family distribution of the True and False Positive hits for TElocal UM at the detection level (cutoff=5 for mouse unstranded (C-D) and stranded (F-G) simulation data. Examination of the False Positives reveals they are largely driven by long young elements, while shorter SINE elements have impaired detection rate.

FC_MM_F - featureCounts using multimappers in “fraction” mode; FC_MM_R - featureCounts using multimappers in “random” mode; FC_UM - featureCounts using unique mappers only; SalTE - SalmonTE; SQuIRE_EM - SQuIRE in EM mode; SQuIRE_UM - SQuIRE in unique mode; TElocl_EM - TElocal in EM mode; TElocal_UM - TElocal in unique mode

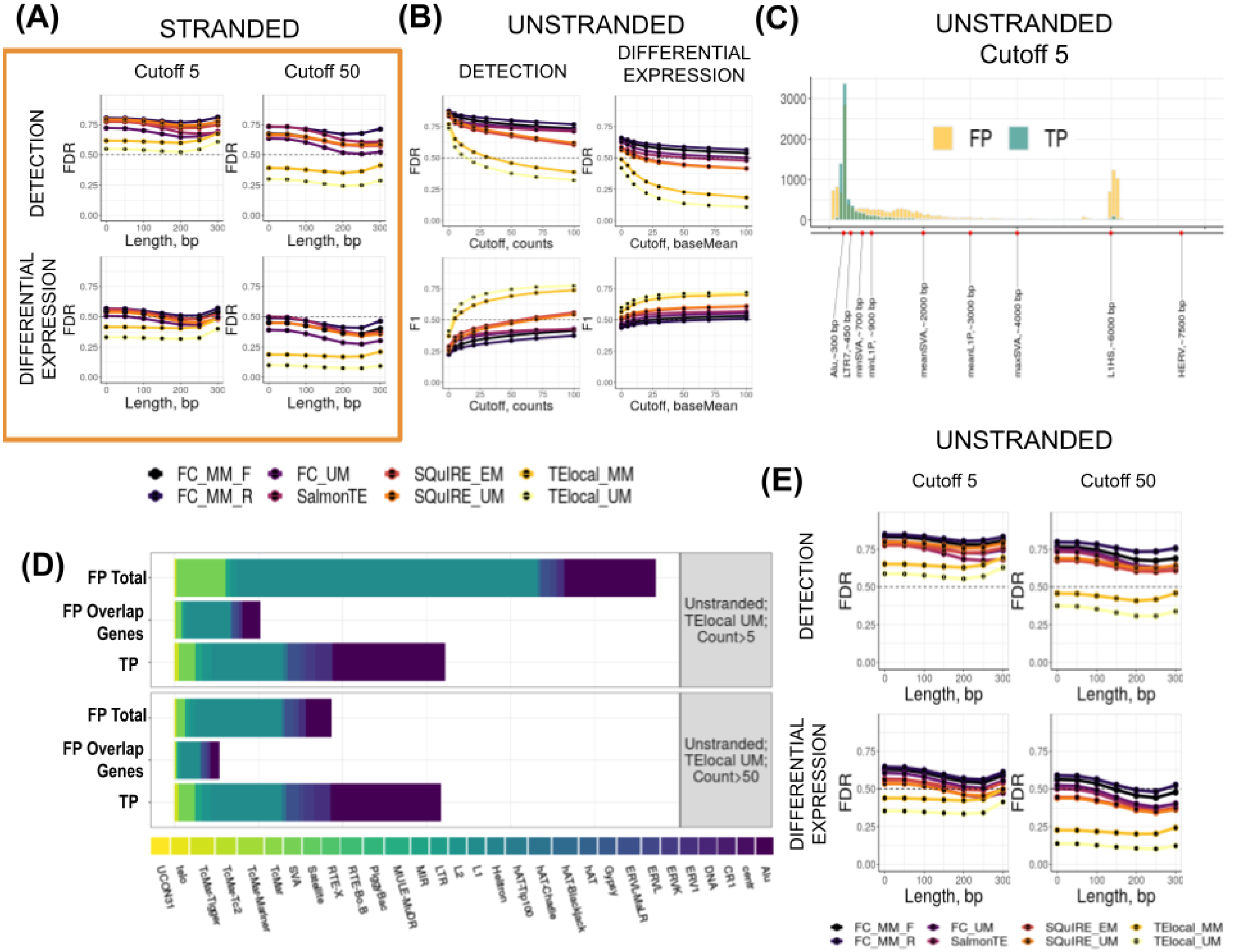

Figure S2. Stranded and unstranded human simulations. (A) - Length Filter applied to Human Stranded Simulation. Exclusion of the shorter elements only minorly improves performance of all strategies, having the most effect on FeatureCounts in UM and SalmonTE. (B) - FDR and F1 scores of the benchmarked tool for detection (left column) and differential expression (right column) analysis at different detection cutoffs for the human unstranded simulated data. All tools perform poorly unless a detection/expression cutoff is applied (raw or baseMean normalized). Such filter allows for a drastic improvement of TElocal performance in particular in the unique mode. However their efficiency is limited. (C) - Length distribution of False and True Positives detected by TElocal_UM at the cutoff 5 for the human unstranded simulated data. While very few L1Hs elements were simulated, multiple false hits were identified for this subfamily, reflected by the secondary peak for FP. (D) - Composition distribution of the True and False Positive elements detected by TElocal UM at the quantification level (cutoff 5 and 50 reads) for the human unstranded simulated data; total FP (FP Total) and FP overlapping stimulated genes (FP Overlap Genes), True Positive (TP). False Positive hits are driven by Alu, L1 and SVA elements, which can be greatly reduced with higher expression/detection filter. Only a minority of False Positive cases can be explained by gene-derived reads. (E) - Application of a length cutoff as a filtering measure to reduce false positive hits for quantification (top row) and differential expression detection (bottom row) at the expression cutoff 5 (left column) and 50 (right column) for the human unstranded simulated data. No significant FDR reduction is achieved with the length filter.

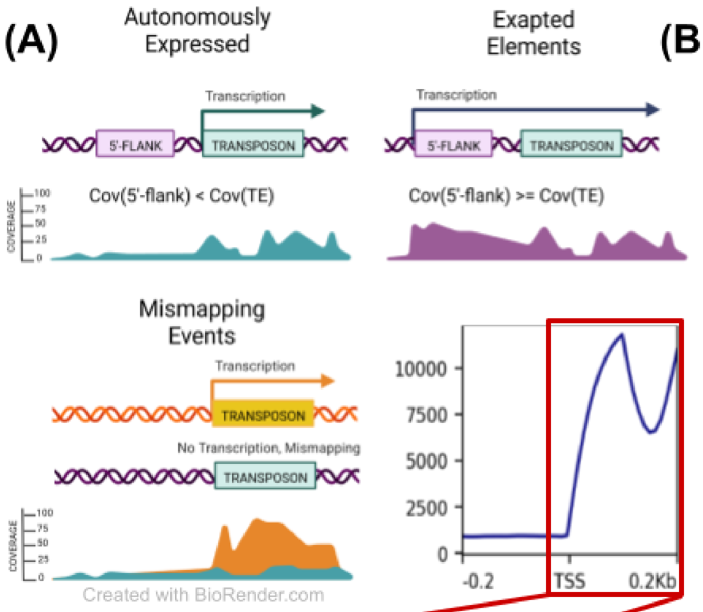

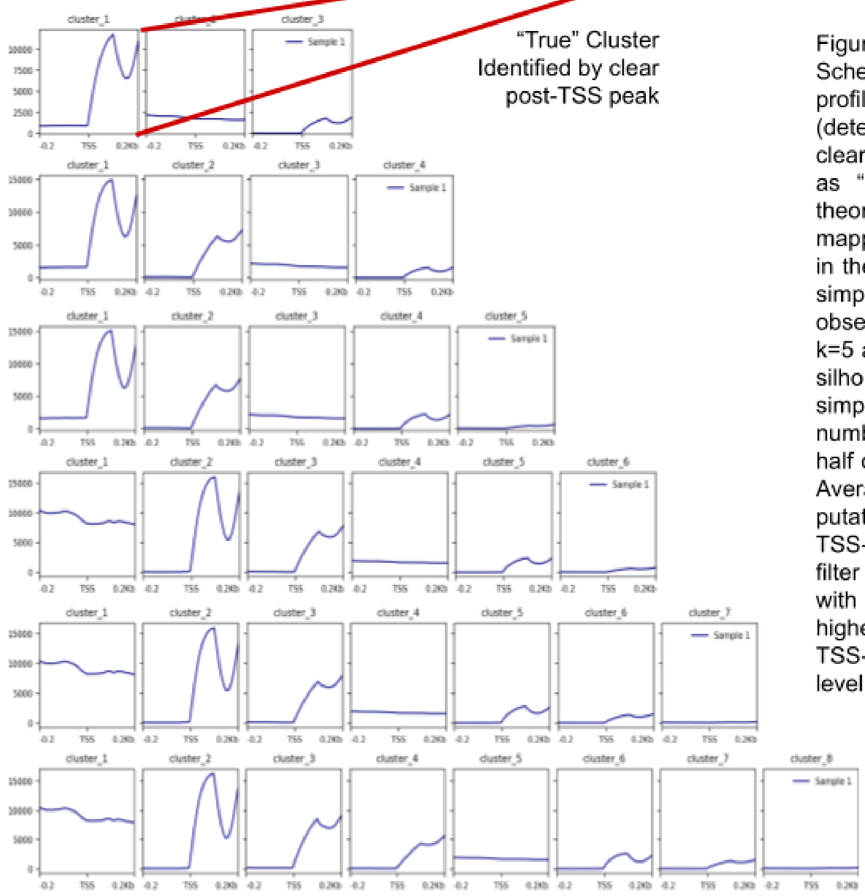

(B)

K	"True" Clusters	TP	FP	FN
k=3	C(1, 3)	2897	7908	5223
k=4	C(1, 2, 4)	2485	9628	5635
k=5	C(1, 2, 4)	7167	1046	953
k=6	C(2, 3, 5)	7032	914	1088
k=7	C(2, 3, 5, 6)	7915	1287	205
k=8	C(2, 3, 4, 6, 7)	7957	1446	163

(C)

Detection Cutoff, counts	TP	FP	FN
5	8120	10348	380
50	7984	3958	516

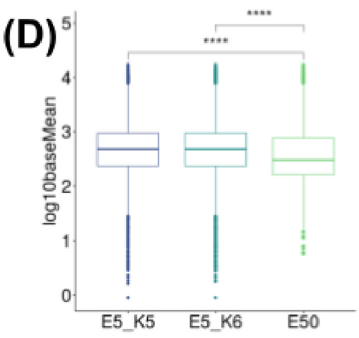

Figure S3. TSS-profiling for human stranded simulation. A - Schematic representation explaining the motivation to use TSS profiling. Example TSS Profiles for k-means clustered TEs (detection cutoff 5) for Sample 1. Only clusters with visually clearly identifiable post-TSS peak in coverage are considered as "True". Clusters with even coverage or pre-TSS peak theoretically represent mostly false hits due to spurious read mapping from reads derived from a related element or inclusion in the longer transcript. B-C Statistics for TSS profiling (B) and simple detection/expression filtering (C) for Sample 1. We observe optimal trade-off for the retained FP and lost FN for k=5 and k=6, which are coincidentally k-values with the highest silhouette scores (Fig.2A). While very few TP are lost with simple detection cutoffs, the number of FP hits exceeds the number of TPs for low detection cutoff and comprises over a half of the total number of TPs for higher expression cutoff. D - Average log10-transformed normalized expression of the putatively differentially expressed TEs as detected with TSS-profiling (E5_K5 and E5_K6) and simple high expression filter (E50). While some lower expression outliers are retained with TSS profiling, average expression for the retained TEs is higher than that detected with simple expression filter. Hence TSS-profiling filters out FP hits irrespective of their expression level.

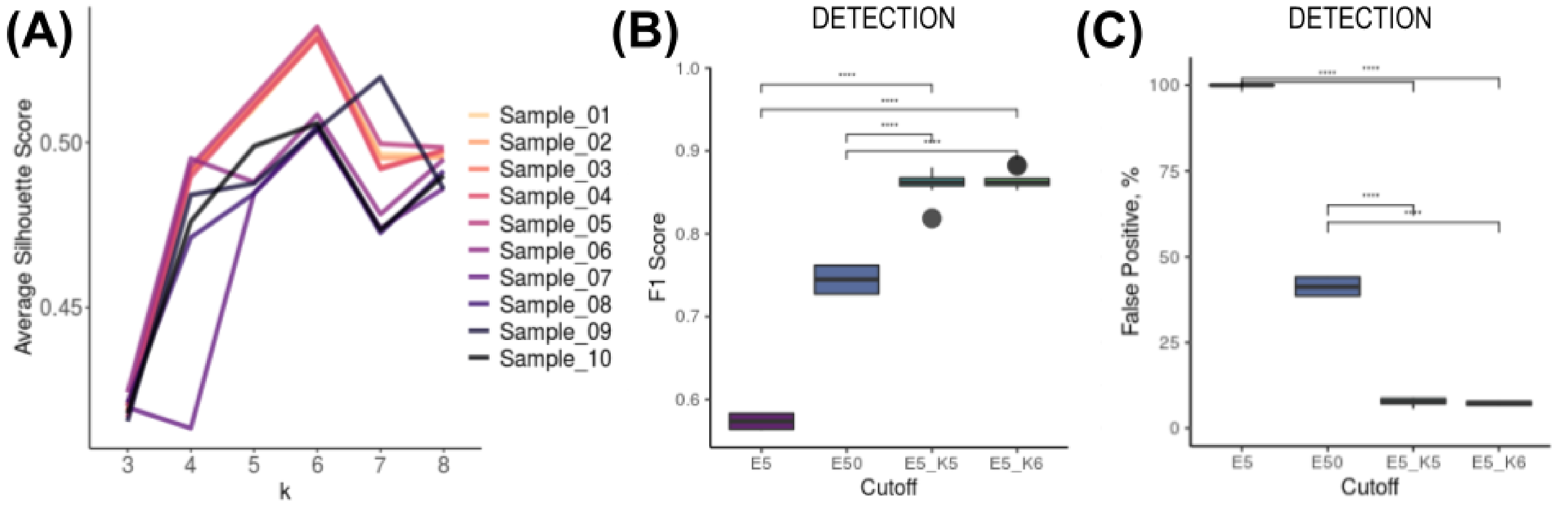

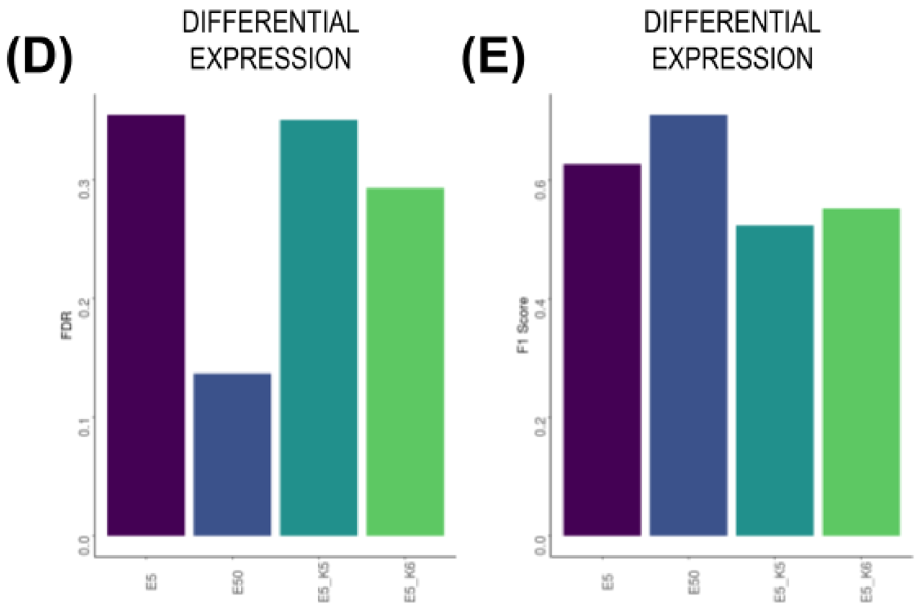

Figure S4. TSS-profiling improves detection for human unstranded simulation, but not the differential expression detection. (A) - Silhouette scores for k-means clustering used during TSS-profiling, plotted per sample. The optimal k varies for samples, but in general achieves its optimum around k=6. (B-C) - F1 Score and False Positive Percentage comparison for simple detection filtering (E5, cutoff 5 counts and E50, cutoff 50 counts per element) and TSS-profiling (E5_K5 and E5_K6, cutoff 5 counts combined with TSS clustering at k=5 and k=6). TSS profiling outperforms both simple detection cutoffs, allowing for lower FP numbers and improvement of overall F1 score. (D-E) - FDR for differential expression detection comparison for simple expression filtering (baseMean 5 and 50, E5 and E50) and TSS profiling (E5_K5, E5_K6). TSS profiling did not improve FDR for differential expression detection as compared to simple detection cutoffs (E5, E50).
TElocal_UM was used to improve upon as the best performing tool.

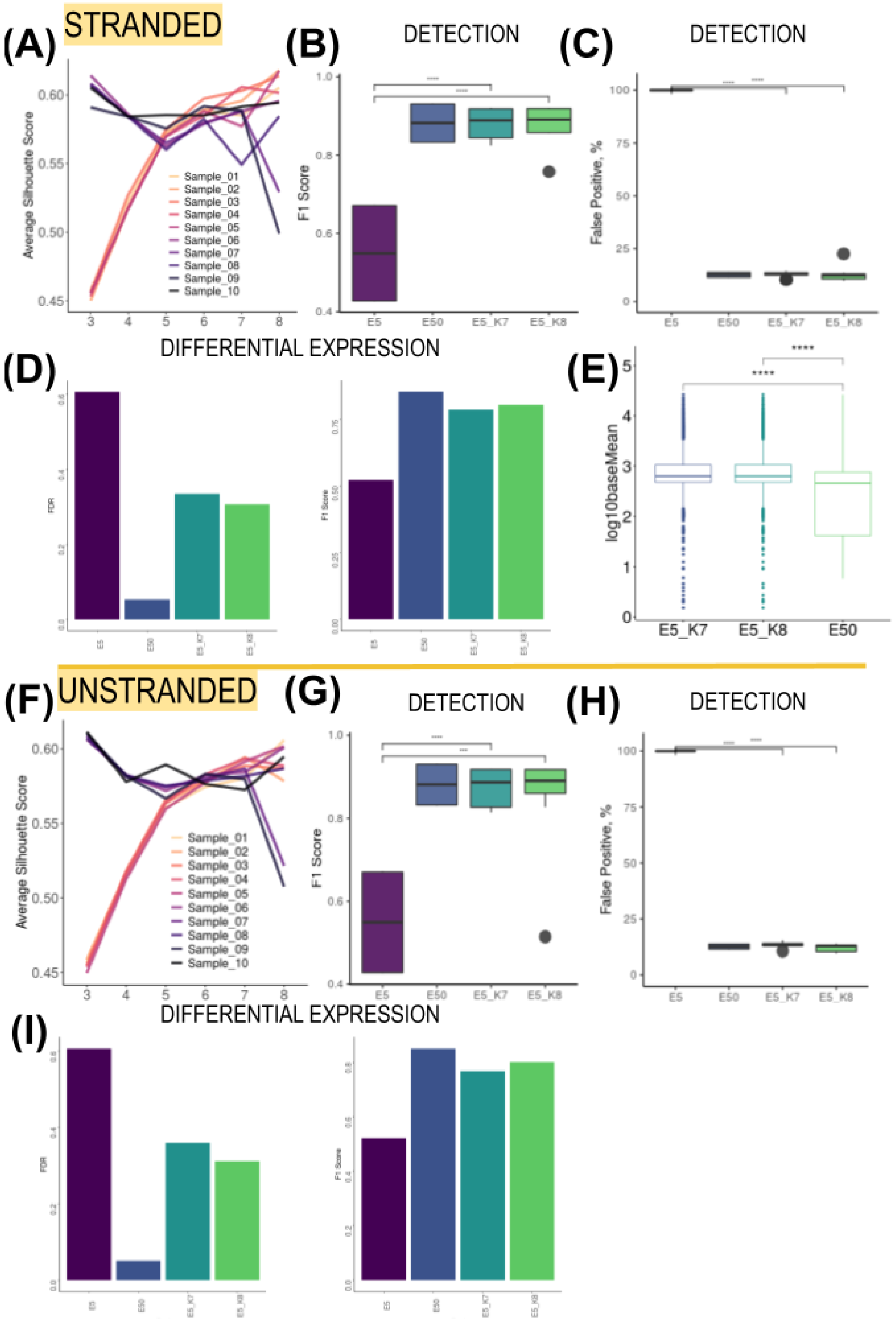

Figure S5. TSS profiling applied to the simulated mouse stranded (A-E) and unstranded (F-I) model. (A, F) - Average silhouette scores for different k values per each sample for stranded (A) and unstranded (F) simulation. Best silhouette scores for most samples are reached with k=7.

(B-C, G-H) - F1 Scores and False Positive Percentages for TE Detection using TElocal UM at Expression Cutoffs 5 and 50, and combination of Cutoff 5 with TSS-profiling (E5_K7, k=7; E5_K8, k=8) for mouse stranded (B-C) and unstranded (G-H) simulation. Both increase in the detection threshold and TSS profiling significantly improve upon F1 Score, and more specifically False Positive numbers. (D, I) - FDR and F1 Scores for Differential Expression Detection using TElocal_UM with TSS profiling for stranded (D) and unstranded (I) simulations. While TSS profiling (E5_K7, E5_K8) improves both FDR and F1 Score as compared to applying low expression cutoff (E5, baseMean 5), higher expression cutoff (E50, baseMean 50) outperformed TSS profiling for the mouse simulations. (E) - Log10-transformed DESeq2 normalized expression of the detected differentially expressed TEs using TSS profiling (E5_K7, E5_K8) compared to filtering with high expression cutoff (E50). TSS profiling does allow for capturing a number of lower expressed TEs, however their average expression is higher when compared to loci retained with expression filtering (E50).
